# Behavioural Models of Risk-Taking in Human–Robot Tactile Interactions

**DOI:** 10.3390/s23104786

**Published:** 2023-05-16

**Authors:** Qiaoqiao Ren, Yuanbo Hou, Dick Botteldooren, Tony Belpaeme

**Affiliations:** 1 AIRO-IDLab, Faculty of Engineering and Architecture, Ghent University-Imec, Technologiepark 126, 9052 Gent, Belgium; tony.belpaeme@ugent.be; 2 WAVES Research Group, Faculty of Engineering and Architecture, Ghent University, Technologiepark 126, 9052 Gent, Belgium; yuanbo.hou@ugent.be (Y.H.); dick.botteldooren@ugent.be (D.B.)

**Keywords:** human–robot tactile interaction, non-verbal interaction, behaviour model, risk-taking behaviour

## Abstract

Touch can have a strong effect on interactions between people, and as such, it is expected to be important to the interactions people have with robots. In an earlier work, we showed that the intensity of tactile interaction with a robot can change how much people are willing to take risks. This study further develops our understanding of the relationship between human risk-taking behaviour, the physiological responses by the user, and the intensity of the tactile interaction with a social robot. We used data collected with physiological sensors during the playing of a risk-taking game (the Balloon Analogue Risk Task, or BART). The results of a mixed-effects model were used as a baseline to predict risk-taking propensity from physiological measures, and these results were further improved through the use of two machine learning techniques—support vector regression (SVR) and multi-input convolutional multihead attention (MCMA)—to achieve low-latency risk-taking behaviour prediction during human–robot tactile interaction. The performance of the models was evaluated based on mean absolute error (MAE), root mean squared error (RMSE), and R squared score ($R^{2}$), which obtained the optimal result with MCMA yielding an MAE of 3.17, an RMSE of 4.38, and an $R^{2}$ of 0.93 compared with the baseline of 10.97 MAE, 14.73 RMSE, and 0.30 $R^{2}$. The results of this study offer new insights into the interplay between physiological data and the intensity of risk-taking behaviour in predicting human risk-taking behaviour during human–robot tactile interactions. This work illustrates that physiological activation and the intensity of tactile interaction play a prominent role in risk processing during human–robot tactile interaction and demonstrates that it is feasible to use human physiological data and behavioural data to predict risk-taking behaviour in human–robot tactile interaction.

## 1. Introduction

Touching an object matters to how we experience, perceive, and relate to that object [1]. It therefore comes as no surprise that touch is an important modality when interacting with robots. The application of human–robot tactile interaction in various domains—such as medicine, rehabilitation, therapy, and education—has received attention in recent years [2,3]. Tactile interaction involves the exchange of information through touch, and it plays a vital role in creating more natural and intuitive interactions between people and robots [4]. Humanoid robots, such as the Aldebaran Robotics’ Nao, have the ability to detect and respond to tactile interactions with users, and touch has been explored as a prime modality to express emotion to a robot [5]. The importance of tactile interaction is evident in various industries where humans need to work alongside robots [6]. Key to this is that robots not only detect a touch event but also are able to sense the intensity, temporal aspects, and spatial configuration of tactile events [7]. With the ability to sense and respond to high-dimensional tactile feedback, social robots can improve their effectiveness, ensure safe interaction with users, and achieve a more natural and intuitive interaction with people.

In this paper, we combine tactile interaction with risk-taking. The study of risk-taking is a multidisciplinary field that draws from psychology, sociology, economics, and other fields. It is widely recognized that risk-taking is a complex behaviour that is influenced by a multitude of factors, including personal characteristics, societal norms, and the perceived benefits and costs of taking a particular risk [8]. Many theories have been proposed to explain risk-taking behaviour, including cognitive, biological, and behavioural perspectives. The study of risk-taking sheds light on human behaviour and decision-making processes and has practical implications for individuals, organizations, and society as a whole [9]. In addition, risk-taking behaviour has been linked to a wide range of negative outcomes such as financial instability, health problems, and social problems [10,11].

There is a growing body of evidence that suggests that the nature of human–robot tactile interaction can significantly influence an individual’s risk-taking behaviour [12]. Despite the safety hazards associated with physical interaction with social robots, several researchers have highlighted the importance of tactile interaction even with the Nao robot, which is the most widely used robot in human–robot Interaction (HRI) studies. For instance, Willemse and colleagues found that gentle and soft touch by a Nao robot can lead to feelings of comfort and safety [13]. Additionally, Zhou and colleagues [14] found that touching the private areas of the Nao robot, such as the buttocks and genital area, leads to a stronger physiological response than touching its more accessible areas, such as the hands and feet. This highlights the importance for designers to consider the potential positive and negative effects of human–robot tactile interaction [15]. Furthermore, researchers have explored the role of affective touch during interactions with the Nao robot. For example, Yohanan and colleagues [16] investigated how humans communicate emotional intent through touch to a small robot—dubbed a “haptic creature”—and describe the gesture patterns that participants use and their expectation of the robot to reciprocate gestures. Andreasson and colleagues [5] explored how people can convey emotions to the Nao robot through touching and found high agreement with human–human affective tactile communication. Thus, while the Nao robot is not explicitly designed for physical interaction and may pose safety risks, its use is still acceptable for tactile interaction in HRI studies, given the significant findings regarding the impact of tactile interaction on the user’s experience and behaviour.

Recent research has suggested that both physiological and psychological factors can influence risk-taking behaviour [17]. Physiological factors such as heart rate, interbeat interval, blood volume pulse, and electrodermal activity have been found to be related to risk-taking behaviour [18]. Psychological factors such as personality traits, cognitive biases, and emotions have also been found to be related to risk-taking behaviour [19]. For example, individuals with higher levels of sensation-seeking, impulsivity, and reward sensitivity tend to engage in riskier behaviour. Cognitive biases, such as overconfidence and optimism bias, can also lead individuals to underestimate the risks associated with certain activities [20]. Negative emotions, such as anxiety or depression, can also influence risk-taking behaviour by altering the perception of the potential gains and losses associated with a particular behaviour [21], which can be reflected through the physiological data.

As risk-taking behaviour is multifaceted, it is perhaps not surprising that touch can have an influence on individual’s inclination for risk-taking and that tactile interaction with robots can influence risk-taking [12]. As tactile interaction is likely to become more important between humans and robots, it is imperative to develop accurate predictive models for individual responses to tactile interaction, in the case of this research, a model of individuals’ risk-taking behaviour. Moreover, it is essential to contextualize risk-taking behaviour to suit different situations. The paper includes the following contributions: (1) an exploration of the quantitative interplay between human–robot tactile interaction, physiological responses, and risk-taking behaviour; and (2) a risk-taking prediction behaviour model with high accuracy, relevant to human–robot tactile interaction, taking physiological responses as inputs and adapting to individuals with varying levels of risk-taking behaviour in real time during human–robot tactile interaction. Research has shown that physiological signals can influence behaviour, especially in situations involving potential risks [22]. Some studies have also examined the relationship between financial risks and physiological data [18] while others have investigated the impact of tactile interactions on risk-taking behaviour [12]. However, this study is the first to integrate tactile intensity with a robot and real-time physiological data to predict risk-taking behaviour with the robot, considering that robots are becoming increasingly integrated into our daily lives.

The following sections describe the experimental design and data collection methodology of the human–robot tactile interaction study. Subsequently, the data processing methodology and performance metrics used in the study are introduced. We used a mixed effect model and compared it to a nonlinear model, which served as a baseline, representing a low-latency risk-taking prediction behaviour model, which uses a convolutional neural network (CNN)–Transformer structure. The paper concludes with a summary of findings and suggestions for future work.

## 2. Data Collection

### 2.1. Participants

We recruited 38 participants (19 males and 19 females) for our study through a social media campaign. The participants, who had a mean age of 27.0 ± 2.2 years, were offered a voucher worth EUR 5 at an online store and were randomly assigned to one of four experimental conditions. We excluded individuals who reported difficulties with reading or hearing, or those with acute or chronic heart conditions that could affect data collection. Additionally, we excluded individuals with a background in robotics.The study and data collection complied with the ethics procedures of Universiteit Gent, and all participants provided informed consent before participating.

### 2.2. Experiment Design

We use data from a prior study in which participants had a tactile interaction with a Nao robot [12]. In this study, the robot encouraged participants to take risks during a computer game. This was used to evaluate whether or not and to what extent tactile interaction with a robot can have an influence on people’s behaviour, and specifically on their risk-taking behaviour. We chose risk-taking behaviour, as it provides a strong and measurable outcome, and earlier work has shown that people’s risk-taking can be influenced by a robot [23]. In the study, we measured participants’ physiological data related to heart rate, blood pulse, skin conductivity, and body temperature. These data were recorded together with participants’ risk-taking in the game.

The dataset contained interbeat interval (IBI) data, electrodermal activity (EDA), skin temperature (TEMP), and blood volume pulse (BVP) as input, and BART scores as the label. We divided the data into three datasets: training, validation, and test sets. These datasets had no overlap or data leakage between them, and we used the same, training, validation, and test dataset for all models (mixed effect model, SVR model, and MCMA model).The data collection process took approximately 70 min on average for each individual. During this time, the participants were exposed to four unique human–robot tactile interactions [12]. The following sections describe the data and the collection process in more detail.

### 2.3. Human–Robot Tactile Interaction Conditions

**Figure 1:**
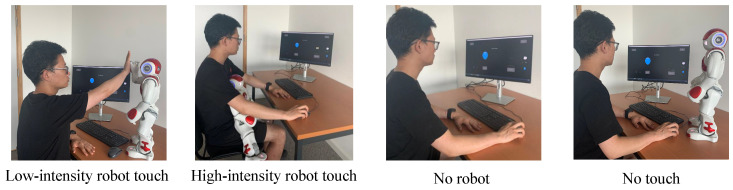
The four interaction conditions used in the study [12].

Participants interacted with an Aldebaran Robotics’ Nao humanoid robot and had either a low-intensity interaction (LI), a high-intensity interaction (HI), no tactile interaction with the robot (NT), or completed a task without the presence of a robot (NR). The study aimed to investigate the influence of tactile interaction on task performance during a BART task by exposing participants to four different conditions in a random order (see Figure 1). The purpose of randomly balancing the order of the conditions was to avoid carryover effects and practice effects. Carryover effects occur when the experience of one condition influences the experience of subsequent conditions, while practice effects occur when participants improve their performance due to repeated exposure to the same condition [24].

In the low-intensity tactile interaction condition (LI), participants were briefly engaged in low-force tactile interactions with the Nao robot at specific moments when they were encouraged to move to the next balloon or to take more risks. The interactions involved giving a high-five, shaking hands, or touching the robot’s head. In contrast, in the high-intensity tactile interaction condition (HI), participants had prolonged and high-force tactile interactions with the robot as it invited them to be taken on the participant’s lap, with both the participant and robot facing the screen together. The robot also used its arms to point and gesticulate. In the no-touch tactile interaction condition (NT), the robot only encouraged participants to take more risks through verbal means, while the no-robot tactile interaction condition (NR) served as a control and baseline for comparison, as no robot was present and participants played the BART game without any encouragement to take more risks. The data on participants’ risk-taking behaviour were extracted from the BART task. Additionally, physiological data were collected from each participant throughout the experiment.

### 2.4. Physiological Data

We used the Empatica E4 sensor to capture physiological data, which was worn on the nondominant wrist of participants across the four experimental conditions. This particular sensor is intended for collecting physiological data and has been employed in earlier studies, which was used to assess various stress proxies, including IBI data, EDA, TEMP, and BVP [18,25]. The E4 wearable sensor has several advantages due to its unique combination of an EDA and a photoplethysmography (PPG) sensor. These sensors allow the E4 to simultaneously measure sympathetic nervous system activity and heart rate, providing researchers with a more comprehensive view of physiological responses. Additionally, the E4 has been shown to provide accurate measurements that are close to those of professional high-end laboratory equipment [26]. Previous research compared three noninvasive wearable devices, and the results showed a significant preference for the wrist-worn devices, with the Empatica E4 device being most often preferred [27]. Another advantage of the E4 is its ability to provide raw unprocessed data, which allows researchers to conduct in-depth studies on the physiological data.

#### Risk-Taking Behaviour Data

The risk-taking behaviour data were collected using the balloon analogue risk task (BART).The BART score is used as a measure of the participant’s risk-taking behaviour.

The BART is a laboratory-based measure of risk-taking that involves participants inflating virtual balloons in exchange for money, with each balloon having a small chance of popping and causing the participant to lose all the money earned from that balloon. The BART is a reliable and validated tool for assessing real-world risk-taking propensity and is commonly used in research [28]. Unlike other common measurements of risk, which typically require historical returns data for an investment or benchmark index, the BART is a behavioural measure that does not require access to private data.

The BART has been shown to be associated with psychological measures of risk-taking and self-reported behavioural risks [29]. The BART has sound experimental properties and risk-taking behaviour on the BART has been correlated with scores on measures of sensation seeking, impulsivity, and deficiencies in behavioural constraint [28].

The BART scores, refs. [28,30] which reflect the number of balloons inflated, can provide insight into an individual’s risk-taking behaviour [28]. The score isthe mean number of pumps each unexploded balloon received. Higher BART score indicates a greater likelihood of engaging in risky behaviour.

## 3. Methodology

As demonstrated in our previous study [12], human–robot tactile interaction can affect and be correlated with human risk-taking behaviour, particularly in relation to different intensities of interaction and stress levels. As a next step, we aimed to investigate the relationship between physiological and risk-taking behaviour, with the ultimate goal of developing a mathematical model that incorporates all relevant factors and can predict human risk-taking behaviour. To achieve this, we propose three behaviour models in this study.

### 3.1. Data Analysis

Data processing and analyses were performed using Python and R. The data was analysed using a mixed effects model, feature selection techniques, machine learning methods, and deep learning methods. The mixed effects model was used to investigate the relationship between physiological and risk-taking behaviour as measured by BART scores. The feature selection techniques and machine learning models were used to identify the most important variables related to BART scores. Along with the mixed effects model, simple linear and nonlinear regression models were used to explore the relationship between the variables and BART scores. Finally, we explored deep learning to achieve end-to-end prediction of risk-taking propensity. To evaluate the performance of the models, we used different evaluation metrics such as $R^{2}$, MAE, and RMSE.

### 3.2. Proposed Models

#### 3.2.1. Physiological Features

From the physiological signals during the human–robot tactile interaction, we constructed a synthetic metric to characterize each participant’s risk-taking behaviour. The extraction of physiological data is explained below.

To quantify each participant’s risk-taking behaviour, we synthesized a metric using the physiological signals collected during human–robot interaction. Four features were calculated from the raw IBI signal to measure activation, including the average number of beats per minute (mHR), average interbeat interval (mRRi), the standard deviation of the interbeat interval ($IBI_{S}DNN$), and the root mean square sum of the successive interbeat interval difference ($IBI_{R}MSSD$). From the raw EDA signal, three features were calculated to measure risk-taking activation, including average skin conductance (mAmp), the average absolute first difference of skin conductance (Slope), and the number of times the EDA signal increased more than 0.05 μs in less than 5 s (event). BVP was measured using the E4 sensor, and three features were calculated to measure activation, including the average absolute value of BVP (mBVP), the minimum value of BVP (miBVP), and the maximum value of BVP (maBVP). Skin temperature was used as a measure of activation, calculated as the instantaneous rate of change in temperature (TCR); these features were inspired by [18].

#### 3.2.2. Multicollinearity

**Figure 2:**
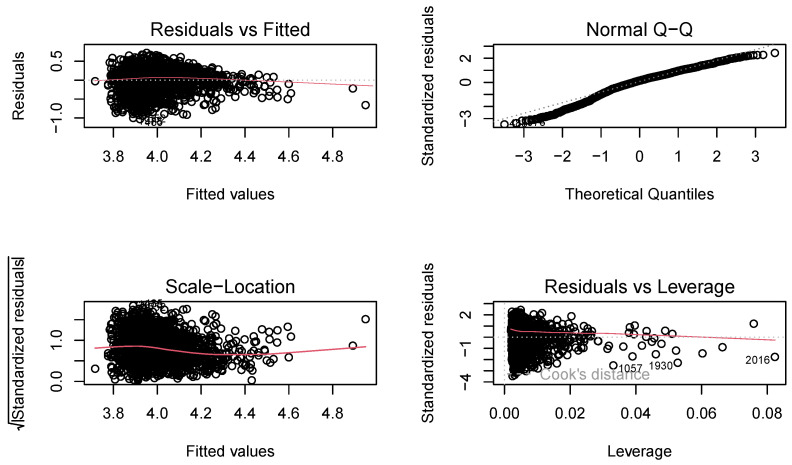
Model diagnostic plots, which consist of four subplots, with the top-left plot titled “Residuals vs. Fitted” used to evaluate the assumption of a linear relationship. The top-right plot titled “Normal Q-Q” was used to test the normality of the residuals. The bottom-left plot titled “Scale-Location (or Spread-Location)” was employed to examine the homogeneity of variance of the residuals. Finally, the bottom-right plot titled “Residuals vs. Leverage” was used to identify influential cases that may significantly impact the regression results when included or excluded from the analysis.

In many physiological experiments, the predictor variables cannot be precisely controlled and thus change together (i.e., they are highly correlated). There is a redundancy of information in the physiological measures, a phenomenon called multicollinearity [31], that leads to numerical problems in estimating the parameters in regression equations; the parameters are often of incorrect magnitude or sign or have large standard errors. Based on this, we built a mixed effects model and conducted a test of multicollinearity. For a cross-sectional set of data containing 2257 observations, the $R^{2}$ value was 0.31. The F-value (19.39) was highly significant ($p<2.2\times 10^{-16}$). implying that all the explanatory variables together could significantly explain the log of risk-taking behaviour. However, regarding the individual regression coefficients, it was seen that two variables (IBI-mHR and IBI-mRRi) were not statistically significant while the EDA-slope was significant only at 0.05 level of significance. The model diagnostic plots are shown in Figure 2, and these were used to assess the assumptions of a linear regression model by examining the residuals in four different ways. The first plot, residuals vs fitted, was used to evaluate the linearity assumption, which appeared as a horizontal line with no discernible patterns, indicating a linear relationship, which is desirable. The second plot, normal Q-Q, which is also referred to as a normal quantile–quantile plot, can be used to test the normality assumption of the residuals. Ideally, the residuals should follow a straight dashed line, and we obtained an approximately straight line, which is acceptable. The third plot, scale–location (or spread–location), can be used to check for the homoscedasticity of the residuals. A horizontal line in the plot with evenly spaced points is indicative of homoscedasticity. The fourth plot, residuals vs. leverage, can be used to identify influential cases, i.e., extreme values that may impact the regression results if included or excluded from the analysis; however, we did not delete any extreme data. Then, we used the Farrar–Glauber test (F-G test) for multicollinearity checked the overall and individual diagnostic checking for multicollinearity, respectively. The standardized determinant was found to be 0.001 while the calculated value of the Chi-square test statistic was found to be 15,651.43; as this was highly significant, the presence of multicollinearity in the model specification was indicated. The F test showed that either the variable. IBI-mHR or IBI-mRRi, could be the root cause of multicollinearity; therefore, we used lasso regression as a regularization method to resolve the problem of multicollinearity. We picked the features for the optimal model tested by lasso regression, which also eliminated variable IBI-mHR. The chosen physiological features for further analyses were mmRRi, SDNN, RMSSD, mAmp, Slope, event, mBVP, miBVP, maBVP, and TCR.

#### 3.2.3. Mixed Effects Model

**Figure 3:**
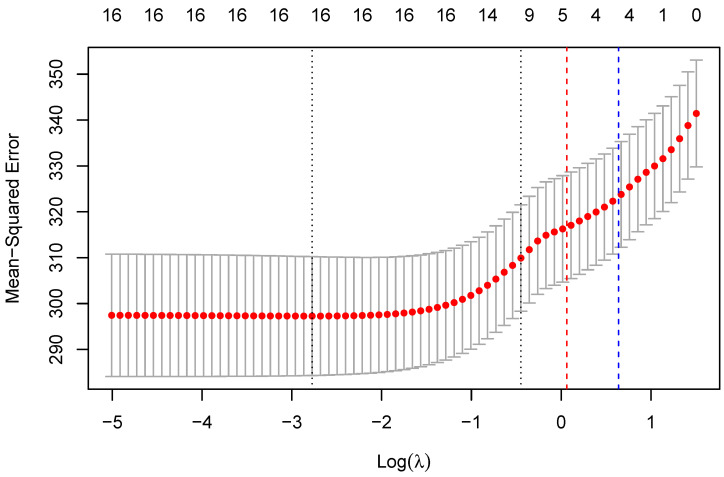
Lasso coefficient path visualization. The red and blue vertical lines on the plot are the values of the minimum lambda and 1-standard-error lambda, respectively, and were obtained from a lasso regression model that had undergone cross-validation.

This study used a hierarchical mixed effects model that accounted for the within-subject design of the data collection and correlation among physiological variables. Lasso regression is a commonly used method for addressing multicollinearity and performing feature selection simultaneously. The idea is to apply lasso regression regularization to the model, which will force some of the coefficients to be exactly zero, effectively removing these variables from the model. The remaining variables with nonzero coefficients can then be used as inputs to the mixed effects model. The features selected by the lasso regression optimal model (mRRi, SDNN, RMSSD, mAmp, Slope, event, minimum value of BVP, maximum value of BVP, and TCR) were fed into the mixed effects model, which did not exhibit multicollinearity with all the variance inflation factors (VIFs) being smaller than two, thus also removing the variable IBI-mHR. The lasso coefficient path visualization can be seen in Figure 3.

The model incorporated physiological data and human–robot tactile intensity as input, with the dependent variable representing behavioural variables (conditions that consist of four classification variables). The random effect for the *j*th observation in the *i*th group, which captures the variation in the intercept and/or slope among the different groups was represented by $u_{i}j$, while coefficients for IBI, EDA, BVP, and TEMP features were represented by $\beta_{0}$ to $\beta_{4}$. While the residual component ϵ represented variability in residuals unexplained by fixed and random effects. Subscript ij means the *j*th observation in the *i*th group. $y_{ij}$ is the response variable (BART scores) for the *j*th observation in the *i*th group. $ibi_{ij}$ is IBI features, $bvp_{ij}$ means BVP features, $eda_{ij}$ represents EDA features, $tcr_{ij}$ means the average change of temperate rate, and $hrtc_{ij}$ indicates the human–robot tactile interaction intensity. $\beta_{0}$ to $\beta_{4}$ typically represent the fixed effect regression coefficients, which represent the average change in the response variable for a unit change in the corresponding predictor variable, assuming all other predictor variables are held constant. These coefficients were estimated from the data and used to make predictions for new observations. In contrast, the random effects in a mixed effects model represents individual deviations from the fixed effects, and they are assumed to follow a certain distribution. By including both fixed and random effects, mixed effects models can account for both systematic variation due to known predictors and random variation due to unobserved or unmeasured factors.

(1) $$\begin{array}{c} y_{ij}=\beta_{0}\cdot ibi_{ij}+\beta_{1}\cdot bvp_{ij}+\beta_{2}\cdot eda_{ij}+\\ \beta_{3}\cdot tcr_{ij}+\beta_{4}\cdot hrtc_{ij}+u_{ij}+\epsilon \end{array}$$

**Figure 4:**
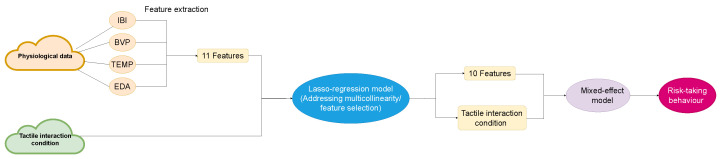
Data flow of mixed effects model.

**Figure 5:**
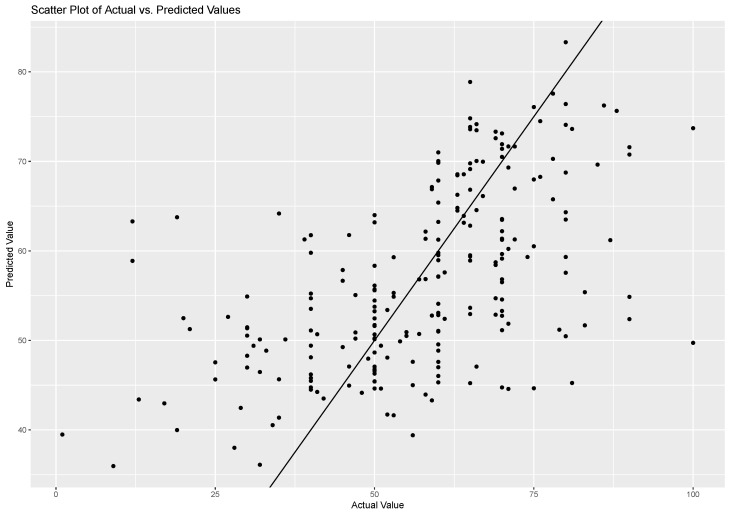
Mixed effects model: actual risk-taking behaviour vs. predicted risk-taking behaviour.

The data flow is outlined in Figure 4. We obtained an RMSE of 14.73, an MAE of 10.97, and an $R^{2}$ of 0.30 on the test dataset. Figure 5 shows the comparison between the scatter plot of actual risk-taking behaviour and predicted risk-taking behaviour. Among the selected features, the TCR was identified as the most significant predictor, followed by $IBI_{SDNN}$, $IBI_{RMSSD}$, $EDA_{slope}$, and tactile interaction intensity. The remaining features were found to have relatively less importance in predicting the target variable. This approach provides a baseline framework for analysing the relationships between physiological and risk-taking behaviour.

### 3.3. Support Vector Regression Model

Initially, we used a mixed effects model as a baseline for analysis. However, the model’s performance on the test dataset was inadequate, as indicated by a high MAE, suggesting a suboptimal model fit. To address this limitation, we explored a nonlinear approach by using SVR with an RBF kernel. SVR constructs hyperplanes or sets of hyperplanes in a high-dimensional space for classification or regression and can handle nonlinearly separable data by using the kernel trick to map data into a higher-dimensional space that allows for linear separation. Moreover, SVR effectively handles high-dimensional data [32].

**Figure 6:**
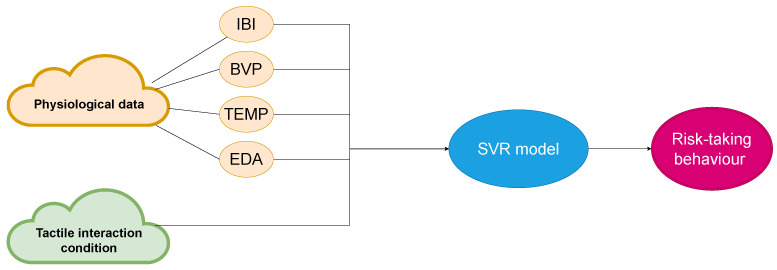
Data flow for SVR model.

To evaluate the effectiveness of the SVR model, we input the original physiological data into the model and assessed the resulting prediction of BART scores using MAE. The SVR model’s performance was only slightly better than that of the mixed effects model, with the data flow shown in Figure 6.

**Figure 7:**
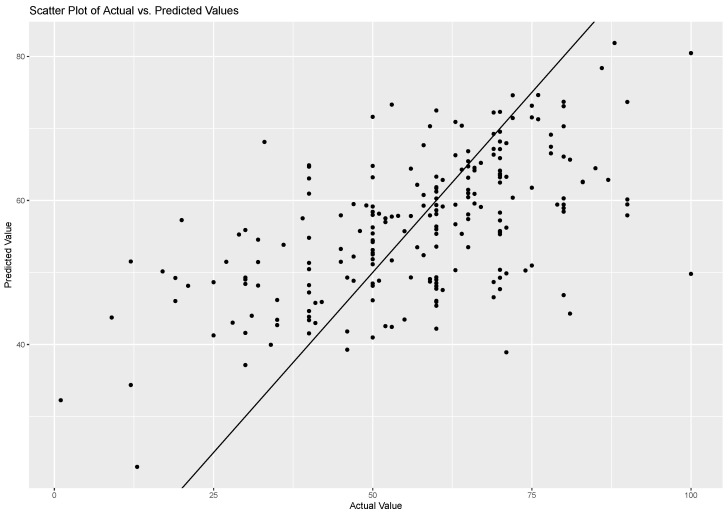
SVR model: actual risk-taking behaviour vs. predicted risk-taking behaviour.

For the SVR model performance, no significant improvement was noted. The test dataset yielded an RMSE of 14.28, an MAE of 10.75, and an $R^{2}$ of 0.35. Figure 7 shows a comparison between the scatter plot of actual risk-taking behaviour and predicted risk-taking behaviour, intercept, and slope from the fitted line, indicating that the data fit better than the did baseline but that the improvement was not substantial. Our analysis of the SVR model’s output, which included all of the input features, revealed consistent results regarding the relative importance of individual predictors. Specifically, the TCR feature exhibited the highest predictive power, followed by $IBI_{SDNN}$, $IBI_{RMSSD}$, $EDA_{slope}$, and tactile interaction intensity. Conversely, the remaining features were deemed to be comparatively less important in the context of predicting risk-taking behaviour. To achieve end-to-end prediction in a shorter time, we shifted our focus to a deep learning model for the next phase of analysis, which has the potential to enhance the accuracy of predictions and reduce the time needed for inference.

### 3.4. The Proposed Multi-Input Convolutional Multihead Attention (Mcma) Model

Deep learning-based models have shown outstanding results in temporal signal processing, leading to the use of the MCMA model in this task. The present section highlights the two key findings. Firstly, our research will delve deeper into the model structure by examining the influence of different neural network architectures and hyperparameters on the predictive performance. Secondly, ablation experiments were conducted on different components of the proposed model to analyse its performance further; specifically, the ablation experiments investigated the efficacy of diverse categories of physiological data. In addition, the impact of integrating attention mechanisms on multiple types of data was analysed. This exploration will provide further insights into the use of attention mechanisms applied to predicting risk-taking behaviour and contribute to advancing the understanding of effective neural network architectures for this task. The study explains the significance of various semantic types of time-series data involved in this task and their importance in achieving better results.

#### 3.4.1. Model Structure

**Figure 8:**
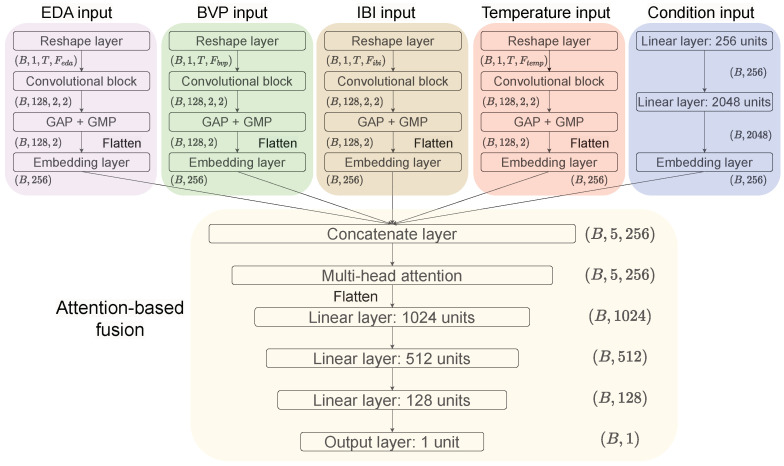
The proposed multi-input convolutional multihead attention (MCMA) model.

For the purpose of achieving real-time risk-taking behaviour without any preprocessing or manual feature extraction, we proposed an MCMA model, and as such implemented an end-to-end predictor when faster behaviour prediction was needed. The raw data of EDA, BVP, IBI and temperature involved in this paper are essentially time series, which vary with different experimental conditions. Given the outstanding feature extraction capability of convolutional neural networks (CNN) in time-series related tasks [33,34] and the excellent performance of the attention mechanism in Transformer [35] in capturing contextual information, this paper proposes an MCMA model, as shown in Figure 8. The proposed MCMA first uses multiple parallel convolutional blocks to extract high-level representations of data with different semantic types, then fuses the learned intermediate representations using multiheaded attention, and finally regresses the target based on the fused information; that is, it predicts the number of clicks by the participants under different conditions.

**Figure 9:**
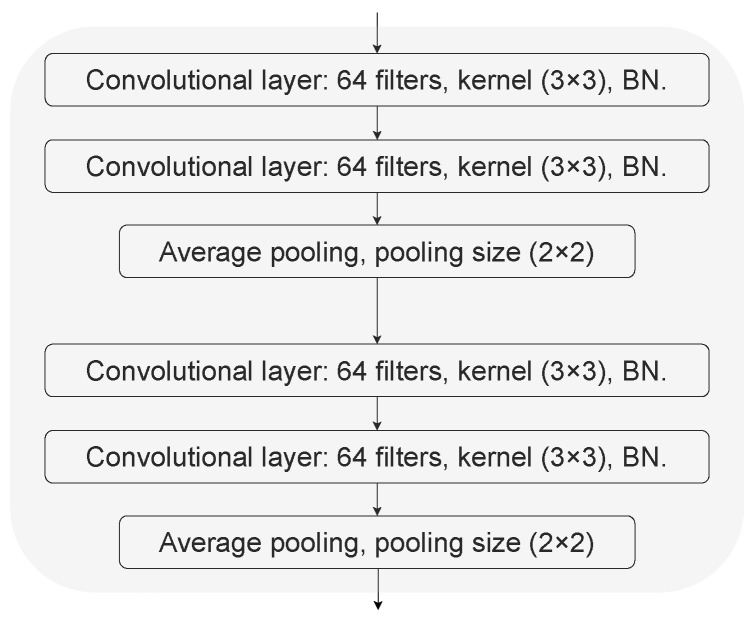
The convolutional block in the proposed MCMA model.

Specifically, the MCMA model in Figure 8 can be divided into two parts: the multi-input branch and the attention-based fusion. For the multi-input branch module, since the raw data of EDA, BVP, IBI and temperature vary in length in time, we chose the mode of each type of sequence data length in each record as its final input length. On the training set used in this paper, the length modes of EDA, BVP, IBI, and temperature were 72, 1200, 16, and 72, respectively. Therefore, the corresponding inputs were (B,72), (B,1200), (B,16), and (B,72), where *B* is the batch size. To extract the features of these time series using the convolutional block in Figure 9, these time series were further sliced into *T* time steps and transformed into a 2-dimensional matrix with *T* rows. The *T* used in this paper is 8, resulting in $F_{eda}$, $F_{bvp}$, $F_{ibi}$, and $F_{temp}$ being 9, 150, 2, and 9, respectively. Note that since $F_{ibi}$ is 2, the pooling size in the convolutional block of the IBI input branch is (2×1) instead of the default (2×2), which retains as much information regarding the data at each time step as possible. In the convolutional layer, batch normalization (BN) was used to prevent model overfitting in training [36]. Following this, the convolution block was used as a pooling layer composed of global average pooling (GAP) and global max pooling (GMP) to downsample the feature map, thus reducing the number of model parameters [37]. To exploit the advantages of GAP and GMP, we summed the averaged and maximized representations. For the condition input branch, this we used one-hot encoding for the 4 class conditions, so its input dimension is (B,4). Raw data with semantic types and input conditions were finally mapped to high-level representation vectors of dimension (B,256) using an embedding layer with 256 units.

Next, the learned representations from multitype inputs need to be fused to generate task-oriented representations. The four types of time series data obtained from the embedding layer and the corresponding condition representation vectors are concatenated into a tensor with dimensions (B,5,256) and fed into the multihead attention (MHA) layer, which is based on the scaled dot-product attention from Transformer [35]. The input of MHA consists of queries of dimension $d_{q}$ and keys of dimension $d_{k}$ as well as values of dimension $d_{v}$, where $d_{q}=d_{k}$ [35]. The attention is calculated on the basis of a set of queries, keys, and values that are packed into matrix Q, K, and V, respectively.

(2) $$Attention\left(Q,K,V\right)=softmax\left(QK^{T}/\sqrt{d_{k}}\right)V$$

Then, the multihead mechanism in MHA facilitates the model to concentrate on representations of different subspaces.

(3) $$\begin{array}{c} MHA\left(Q,K,V\right)=Concat\left(head_{1},\cdots ,head_{h}\right)W^{O},\\ where\;head_{i}=Attention\left({QW}_{i}^{Q},{KW}_{i}^{K},{VW}_{i}^{V}\right),i\in \left[1,h\right] \end{array}$$

where $head_{i}$ denotes the *i*-th attention head. $W_{i}^{Q}$, $W_{i}^{K}$, $W_{i}^{V}$ and $W^{O}$ are the corresponding weights that can be updated in training. For the parameters in MHA (such as $d_{k}$, $d_{v}$ and *h*, etc.), they all follow the default settings of Transformer [35]. The attention in the MCMA model calculates the similarity of each type of data to other types of data and reconstructs the current data based on their similarity to other types of data. Therefore, data of different semantic types under different conditions will automatically align with each other and contain information about the conditions and other semantic time series after undergoing attention-based fusion. In addition, the multihead mechanism in MHA will play a role similar to the filters in the convolutional block and provide a variety of perspectives for cross-type information fusion, which allows the model to focus better on different types of information in the latent space and capture richer features.

Finally, the representations after attention-based fusion are fed into the following linear layers to fit the corresponding target to complete the regression task proposed in this paper, which performs the prediction of BART scores and can be used to predict the risk-taking behaviour based on multiple types of semantic time series under different conditions.

#### 3.4.2. Ablation Study of the Proposed Mcma Model Components

We aimed to investigate the impact of different types of physiological data and tactile interaction intensity on predicting risk-taking behaviour. To achieve this, we conducted an ablation study that involved varying the inputs to the model by excluding different types of physiological data and tactile interaction intensity. The results can be seen in Table 1. We also explored the effect of MHA-based fusion, a popular attention mechanism in deep learning, on the model’s performance. Our findings indicate that the inclusion of all types of physiological data and tactile interaction conditions in the input, along with MHA-based fusion, is the most effective approach for predicting risk-taking behaviour. This highlights the importance of considering the tactile interaction intensity when developing models for predicting risk-taking behaviour. Moreover, our findings suggest that temperature is a surprisingly influential factor for accurately predicting the target variable. Specifically, we observed that the model’s performance metrics, namely the MAE of 4.42, RMSE of 5.9, and $R^{2}$ of 0.88, were significantly impacted by temperature despite it exhibiting poor performance in the ablation study. As such, temperature emerged as the most significant predictor, highlighting its crucial role in accurately forecasting the target variable. Furthermore, our findings indicate that the intensity of tactile interaction during human–robot interaction plays a critical role in predicting risk-taking behaviour. Notably, human–robot tactile intensity exhibited the second-worst performance in the ablation study, with an MAE of 3.94, an RMSE of 5.43, and an $R^{2}$ of 0.89. Thus, our results demonstrate that tactile interaction intensity is a significant predictor of the target variable and cannot be overlooked in accurately forecasting risk-taking behaviour.

Furthermore, we found that excluding any one of the physiological data types resulted in a significant decrease in model performance. This highlights the importance of incorporating all types of physiological data when predicting risk-taking behaviour. Additionally, we assessed the impact of MHA-based fusion on the model’s performance and found that incorporating MHA-based fusion resulted in better model performance compared to models without this attention mechanism. Our findings emphasize the importance of considering all types of physiological data and tactile interaction intensity and incorporating MHA-based fusion to improve the model’s performance.

## 4. Discussion

### 4.1. Experiment Results

Research on risk-taking remains an important area of study with practical implications for individuals, organizations, and society as a whole [38,39]. Negative consequences of risk-taking behaviour can include physical, emotional, and social harm, while positive risks can lead to productive interactions [40]. In the context of human–robot tactile interaction and understanding the impact of tactile feedback, the intensity of tactile interaction on risk-taking behaviour is crucial. By manipulating the level and quality of tactile interactions, it may be possible to regulate risk-taking behaviour and improve outcomes. However, further research is needed to develop more sophisticated models for predicting and influencing risk-taking behaviour in human–robot interactions.

The present study aimed to investigate the relationship between physiological and risk-taking behaviour, as measured by the BART scores. To achieve this, the study collected various physiological data, including temperature, interbeat interval, blood volume pulse, and electrodermal activity. A mixed effects model was employed to determine the correlation between the physiological data and risk-taking behaviour. This is a statistical model used to account for variations between subjects or groups, allowing for a more accurate analysis of the data. Our results showed that physiological data were related to risk-taking behaviour as measured by BART scores. The mixed effects model revealed that heart rate, interbeat interval, blood volume pulse, and electrodermal activity were positively related to BART scores. The implications of these findings are that physiological factors can be used to predict risk-taking behaviour as measured by BART scores. The study also explored the nonlinear methodology SVR model, which is slightly better than the mixed effects model that served as the baseline, in order to obtain a low-latency model with better model performance. An MCMA model was proposed and evaluated using MAE, RMSE, and $R^{2}$ as the evaluation metrics.

The optimal model was found to be the MCMA model, which achieved an MAE of 3.17, RMSE of 4.38, and $R^{2}$ of 0.93. These metrics indicate that the model’s predictions are highly accurate and explain a large proportion of the variance in the data. In comparison, the mixed effects model achieved a baseline MAE of 10.97, RMSE of 14.73, and $R^{2}$ of 0.93, which can be observed in Table 2. This highlights the superiority of the proposed model in predicting risk-taking behaviour. The proposed model can be useful in predicting risk-taking behaviour in 8 s (the shortest input length for EDA, BVP, and IBI are 32, 32, and 8 respectively) with the E4 sensor sampling rate. With a higher sampling rate, a shorter prediction time can be achieved, making it an efficient tool for researchers in understanding the underlying mechanisms of risky decision-making. Based on this model, we also explored the ablation experiments, which indicated the importance of different intensity tactile interaction and various types of physiological data on the risk-taking behaviour prediction. It has been established that the ability to predict risk-taking behaviour is crucial and requires a tailored approach for various contexts. One possible strategy is to modify the level of human–robot tactile engagement, which can have a significant impact on positive and negative risk-taking tendencies. Such adaptive measures can effectively regulate risk-taking behaviour and improve outcomes in diverse situations.

### 4.2. Limitations and Implications

However, this study has some limitations. Firstly, the sample size was relatively small, which might affect the generalizability of the findings. Secondly, only a limited number of physiological factors were considered, and future research should explore other factors that may relate to risk-taking behaviour. Moreover, as participants’ gender and age were collected during the study, we could use these variables in the model. Earlier research has indicated that male and young drivers between the ages of 17 to 29 years have a higher likelihood of having at least one car crash compared to their female drivers and drivers above 50 [41], showing that age and gender have marked predictive power. As our participant sample is not comprehensive, this may introduce a bias or confounds that can affect the accuracy and validity of the predictions. Our results may not be generalizable to all age groups or different populations.

It should also be noted that tactile interaction adheres to cultural norms and that these can rapidly change. The recent COVID-19 pandemic showed that for a brief period, physical interaction was not only impossible but also changed in response to evolving cultural norms governing interpersonal interaction [42]. The observations from this study should be taken in this light: they provide a current snapshot of the cultural norms of the interaction with robots.

Further research should examine the relationship between physiological factors and risk-taking behaviour in different populations and domains [43]. Moreover, it would be worthwhile to explore the neural mechanisms underlying the association between physiological and psychological factors and risk-taking behaviour. Additionally, personality traits could affect risk-taking behaviour and should be investigated in future studies [44].

In addition, the use of the Nao robot as a research tool also presents some limitations. While the robot’s “cute” factor may be appealing to some participants, its touch control capabilities are limited and may not be sufficient for more sophisticated research on human–robot interaction. Future studies could explore the use of other robots or more advanced haptic devices to expand the range of tactile interactions that can be studied.

The study’s findings have several implications for the field of robotics and human–robot interaction. First, the study highlights the importance of touch in human–robot interactions and suggests that physiological and behavioural data can be used to predict risk-taking behaviour in these interactions. This knowledge can inform the design of more effective and intuitive robots that can interact with humans in more natural and meaningful ways. Second, the study’s methods and results could have applications in various domains, such as healthcare, education, and entertainment. For example, the findings could be used to develop more effective therapeutic robots that can help individuals with anxiety disorders or phobias overcome their fears in a controlled and safe environment. Similarly, the study’s methods could be applied to educational settings to help students learn through interactive and engaging human–robot interactions.

Third, the legal and regulatory aspects of using robots in human–robot interactions should be considered. As robots become more ubiquitous in our daily lives, there may be ethical and legal concerns regarding their use, particularly in areas where personal and private data are needed for the robot’s functioning. Tactile data are personal and often considered private and as such warrant the same protection given to other sensitive and personal data.

### 4.3. Summary

In summary, this study aimed to investigate the relationship between physiological factors, the intensity of human–robot tactile interactions, and risk-taking behaviour as measured by BART scores. A mixed effects model was employed as a baseline, and a nonlinear SVR model was explored to predict risk-taking behaviour. A proposed MCMA model outperformed the baseline model in predicting risk-taking behaviour, providing an efficient and accurate tool for researchers studying risky decision-making. We used ablation experiments, which indicated the significance of different intensities of tactile interactions and various types of physiological data on risk-taking behaviour prediction. The findings suggest that adaptive measures, such as modifying the level of human–robot tactile engagement, can effectively regulate risk-taking behaviour and improve outcomes in diverse situations. The results contribute to our understanding of human–robot tactile intensity and physiological data on risk-taking behaviour and could aid in enhancing future risk prediction studies. Risk-taking behaviour can have positive and negative consequences, leading to personal growth, new opportunities, improved decision-making skills, increased creativity, greater rewards or failure, reputation damage, emotional stress, and physical danger [45]. By changing the tactile intensity with which users interact with the robot and monitoring the real-time physiological data, we could adapt people’s risk-taking behaviour on different occasions.

## Figures and Tables

**Table 1:** Ablation study on different types of data and MHA-based fusion.

#	EDA	BVP	IBI	Temperature	Condition	MHA	MAE	RMSE	$R^{2}$
1	✗	✓	✓	✓	✓	✓	3.626	5.016	0.906
2	✓	✗	✓	✓	✓	✓	3.563	4.520	0.928
3	✓	✓	✗	✓	✓	✓	3.867	5.137	0.901
4	✓	✓	✓	✗	✓	✓	4.415	5.940	0.876
5	✓	✓	✓	✓	✗	✓	3.938	5.427	0.887
6	✓	✓	✓	✓	✓	✗	3.882	5.334	0.897
7	✔	✔	✔	✔	✔	✔	**3.174**	**4.377**	**0.930**

**Table 2:** Performance comparison of different models.

Models	MAE	RMSE	$R^{2}$
Mixed effects model	10.97	14.73	0.30
SVM model	10.75	14.28	0.35
Multi-input CNN–Transformer model	3.17	4.38	0.93

## Data Availability

The data presented in this study is available solely for research purposes and can be obtained upon request from the corresponding author.
